# Community-Engaged Research Ethics Training (CERET): developing accessible and relevant research ethics training for community-based participatory research with people with lived and living experience using illicit drugs and harm reduction workers

**DOI:** 10.1186/s12954-023-00818-6

**Published:** 2023-07-06

**Authors:** Jeffrey Morgan, Scott D. Neufeld, Heather Holroyd, Jean Ruiz, Tara Taylor, Seonaid Nolan, Stephanie Glegg

**Affiliations:** 1 School of Population and Public Health, University of British Columbia, 2206 East Mall, Vancouver, BC V6T 1Z3 Canada; 2 BC Centre on Substance Use, 400-1045 Howe St., Vancouver, BC V6Z 2A9 Canada; 3 Department of Psychology, Brock University, 1812 Sir Isaac Brock Way, St. Catharines, ON L2S 3A1 Canada; 4 University of British Columbia, Learning Exchange, 612 Main St, Vancouver, BC V6A 2V3 Canada; 5 University of British Columbia, Office of Research Ethics, 6190 Agronomy Rd., Vancouver, BC V6T 1Z3 Canada; 6 Overdose Prevention Society, 390 Columbia St., Vancouver, BC V6A 4J1 Canada; 7 SpencerCreo Foundation, 610 Main St, Vancouver, BC V6A 2V3 Canada; 8 Department of Medicine, University of British Columbia, 2775 Laurel Street, Vancouver, BC V5Z 1M9 Canada; 9 Occupational Science and Occupational Therapy, University of British Columbia, T325-2211 Westbrook Mall, Vancouver, BC V6T 2B5 Canada

**Keywords:** Research ethics, Community-based participatory research, Harm reduction, People who use drugs, Rresearch ethics pedagogy

## Abstract

**Background:**

Community-based participatory research (CBPR) can directly involve non-academic community members in the research process. Existing resources for research ethics training can be inaccessible to team members without an academic background and do not attend to the full spectrum of ethical issues that arise through community-engaged research practices. We detail an approach to capacity building and training in research ethics in the context of CBPR with people who use(d) illicit drugs and harm reduction workers in Vancouver’s Downtown Eastside neighborhood.

**Methods:**

A project team comprised of academic and community experts in CBPR, research ethics, and harm reduction met over five months to develop the Community-Engaged Research Ethics Training (CERET). The group distilled key principles and content from federal research ethics guidelines in Canada, and developed case examples to situate the principles in the context of research with people who use(d) illicit drugs and harm reduction workers. In addition to content related to federal ethics guidelines, the study team integrated additional content related to ethical issues that arise through community-based research, and ethical principles for research in the Downtown Eastside. Workshops were evaluated using a pre-post questionnaire with attendees.

**Results:**

Over the course of six weeks in January–February 2020, we delivered three in-person workshops for twelve attendees, most of whom were onboarding as peer research assistants with a community-based research project. Workshops were structured around key principles of research ethics: respect for persons, concern for welfare, and justice. The discussion-based format we deployed allowed for the bi-directional exchange of information between facilitators and attendees. Evaluation results suggest the CERET approach was effective, and attendees gained confidence and familiarity with workshop content across learning objectives.

**Conclusions:**

The CERET initiative offers an accessible approach to fulfill institutional requirements while building capacity in research ethics for people who use(d) drugs and harm reduction workers. This approach recognizes community members as partners in ethical decision making throughout the research process and is aligned with values of CBPR. Building capacity around intrinsic and extrinsic dimensions of research ethics can prepare all study team members to attend to ethical issues that arise from CBPR.

## Introduction

Community members in a wide array of “heavily researched communities” regularly experience research as an extractive, exploitative, and harmful practice [1–3]. One key response has been the wider adoption of community-based participatory research (CBPR) approaches, and an international movement toward the greater inclusion of people with lived and living experience with conditions that regularly attract researcher interest (e.g., poverty, substance use, mental health, HIV, etc.). This movement is often summarized as an ethic of “nothing about us without us” [4]. Employing non-academic community members as “peer research assistants” is one strategy for involving people with lived and living experience in CBPR [1, 5, 6] While the development of approaches in the realm of CBPR has increased the involvement and, at times, empowerment of non-academic community members in knowledge production, one key arena remains almost exclusively the purview of academic experts and institutional gatekeepers: research ethics.

“Research ethics” is commonly understood as navigating institutional approval processes and adhering to guidelines for human subject protections, such as those outlined in the Belmont report [7], or Tri-Council Policy Statement: Ethical Conduct for Research Involving Humans (TCPS2) [8]. Many academic institutions require that study team members who interact with research participants or data receive training in research ethics, usually documented by a certificate after completing a standardized, online, and self-administered tutorial [9]. For example, completion of the “Course on Research Ethics” (CORE) tutorial, offered by the Canadian Panel on Research Ethics, is commonly held as an institutional requirement in Canada for study team members listed in the research ethics application [9, 10]. The Collaborative Institute Training Initiative (CITI Program) is another online training and certification resource commonly accepted in the United States (US) and Canada [11, 12]. These training resources are overwhelmingly biomedical in focus (i.e., emphasizing ethical concerns at the individual, rather than community level), and geared toward academic researchers, students, and members of research ethics boards (REBs) (i.e., institutional review boards) [13, 14]. For example, the CITI Program tutorial has been critiqued for being “cumbersome, inaccessible” and “not designed with community research partners in mind” [12].

In the context of CBPR, peer research assistants may face ethical decisions “in the field” and confront moral issues in their research practice [15–17], such as during participant recruitment and data collection. Some of these ethical issues may also be unique to their community experience. For example, peer research assistants may hold dual relationships with research participants (e.g., peers, co-workers), which can pose challenges for obtaining consent, protecting entrusted information, and ensuring integrity of the data collected [18, 19]. Given this context, accessible and relevant research ethics training for all study team members is essential for ethical and inclusive CBPR [12].

To address the limitations of standardized tutorials, a variety of approaches to research ethics training have been developed across different research contexts. For example, Jetter et al. [20] supplemented a self-administered research ethics course designed for university researchers with a two-part “round table seminar” to discuss the content of the Belmont report and highlight issues of particular relevance to American Indian/Alaskan Native communities. Yonas et al. [12] created flexible research ethics training resources for academic and community partners that can be delivered in-person or online and have been deployed in a wide variety of CBPR contexts in the US. Building on these contextualized and tailored accounts in the US, in this article, we present a methodology for the development and implementation of a relevant and accessible approach to research ethics training tailored to CBPR with people who use(d) illicit drugs and harm reduction workers in Vancouver, Canada, which we refer to as the Community-Engaged Research Ethics Training (CERET) initiative:

### The CERET initiative

The CERET initiative is part of an ongoing effort to build capacity around research ethics in Vancouver’s Downtown Eastside (DTES), a neighborhood characterized by impoverishment, resilience, social activism, multiple public health epidemics, and subjected to a disproportionate amount of research [1, 21]. Previous work building research ethics capacity in the DTES has been described elsewhere [2, 22, 23]. In 2018, university researchers and community members participated in a series workshops on the harms and benefits of research in the DTES neighborhood. This work was summarized in a shared vision of community ethics for respectful and responsive research practices, entitled: *Research 101: A Manifesto for Ethical Research in Vancouver’s Downtown Eastside* [24].

The impetus for the CERET initiative arose from the Overdose Prevention Peer Research Assistant'' (OPPRA) project, a CBPR study about the resilience, stability, and wellbeing of frontline harm reduction workers. The OPPRA study engaged frontline harm reduction workers and/or people who use(d) illicit drugs as peer research assistants and was led collaboratively by representatives from the Overdose Prevention Society, and academic researchers affiliated with the University of British Columbia and British Columbia Centre on Substance Use. Through weekly meetings and capacity-building workshops, OPPRA members were involved at every step of the research process, including study design, participant recruitment, data gathering, analysis, and knowledge translation.

Training in research ethics was identified as an early priority for the OPPRA project, as both an institutional requirement and an important capacity-building activity. We found that the existing research ethics training resources, specifically the CORE tutorial, did not meet the needs of our study. Many OPPRA members had limited access to the internet or computer devices, which made completing the standard online CORE tutorial difficult or infeasible. Furthermore, the language and jargon used in the modules were inaccessible to team members without an academic background.

In addition to accessibility issues, we found that existing resources did not reflect the ethical issues most relevant to CBPR, peer research assistants, or the unique context of the DTES [24]. Finally, it was important to the OPPRA team that capacity building around research ethics promoted dialog and the bi-directional exchange of knowledge, including honoring the lived experience of many OPPRA members as previous participants (or “subjects”) of research in the DTES, including their experiences of (un)ethical research practices. The development, implementation, and evaluation of the CERET workshops are described below.

## Methods

### Workshop design

To develop the CERET workshops, university-affiliated researchers, staff from a university-affiliated REB, staff from a university unit focused on community engagement, and a community leader and expert in harm reduction met regularly over the course of five months with a shared goal to develop an accessible and relevant research ethics training. The knowledge exchange between diverse team members was integral to the development of the CERET workshops.

Workshops were designed to align with guidelines for the ethical conduct of research in Canada, outlined in the TCPS2 [8]. Drawing from our respective expertise, the CERET team attempted to identify and distill the most relevant concepts from the TCPS2 and CORE tutorial that was most applicable to CBPR and behavioral (i.e., not clinical or genetic) research. As a result, some chapters of the TCPS2 were de-emphasized or excluded (e.g., Ch. 12, 13), while others were given greater emphasis (e.g., Ch. 9, 10). Workshops were structured around the TCPS2’s three “core principles”: “Respect for Persons”; “Concern for Welfare”; and “Justice.” [8].

To tailor the training to the local context of the DTES, the group augmented content from the TCPS2 by integrating material from the *Research 101 Manifesto* [24]. We also drew from personal experiences navigating ethical issues in our own research to develop and integrate content related to ethical issues that can arise during CBPR, the involvement of peer research assistants, and research with people who use(d) illicit drugs. Workshops used real and fictitious case studies based on research in the DTES to promote discussion, and to provide practical examples of ethical decision-making “in the field.”

The CERET initiative was delivered over six weeks (January–February 2020), as three, three-hour in-person workshops, each with a thirty-minute break. Each workshop was dedicated to one of the three core TCPS2 principles (“Respect for Persons,” “Concern for Welfare,” and “Justice”). The workshops were held at an accessible location in the DTES. Attendees were provided an honorarium of $40, coffee, and lunch for their participation in each workshop.

### Workshop evaluation

Before and after each workshop, attendees self-reported their confidence and familiarity with the learning objectives/content on a 5-point Likert scale, with anchors on 1 (not at all confident) and 5 (extremely confident). Average item rating, and absolute difference, for each learning objective in the pre- and post-workshop period was computed. The lowest and highest score (i.e., range) for each learning objective pre- and post-workshop period is presented.

## Results

Nine OPPRA members and four *Research 101 Manifesto* co-authors attended the three CERET workshops. All attendees had lived or living experience using illicit drugs and/or in frontline harm reduction. The following sections describe the topical focus and approach used in each workshop. Relevant workshop content that corresponds with the TCPS2 is noted by the chapter where applicable. Learning objectives of each workshop, and findings from the workshop evaluation, are presented in Table 1.

**Table 1:** Familiarity with workshop learning objectives content pre- and post-workshop, and absolute difference

Workshop	Learning objectives	Average score pre-workshop (range)^a^	Average score post-workshop (range)^b^	Absolute difference in mean scores for pre- and post-workshop
Workshop 1: respect for persons	1. Explain the core principles and guidelines for ethical research in the TCPS2	1.9 (1–3)	3.7 (3–5)	1.8
	2. Explain the role of an REB	2.5 (1–4)	4.2 (3–5)	1.7
	3. Identify possible risks of research to individuals and communities	3.0 (1–4)	4.3 (3–5)	1.3
	4. Describe the components of informed consent	2.7 (1–4)	3.7 (1–5)	1.0
Workshop 2: concern for welfare	1. Explain the concept of concern for welfare	3.3 (1–5)	4.5 (3–5)	1.2
	2. Identify strategies to maintain confidentiality	2.9 (1–5)	4.3 (3–5)	1.4
	3. Understand the importance of including people with lived and living experience in the research process	3.6 (1–5)	4.6 (3–5)	1.0
	4. Explain the four components of ethical research in the DTES, as outlined in the *Research 101 Manifesto*	3.3 (1–5)	4.2 (3–5)	0.9
Workshop 3: justice	1. Explain the importance of fairness and equity in research	2.8 (1–4)	3.7 (2–5)	0.9
	2. Identify factors that contribute to participant vulnerability	2.7 (1–4)	4.1 (3–5)	1.4
	3. Understand the concept of dual relationships and how disclosing conflicts of interest is important for conducting ethical research	3.1 (1.4)	4.2 (2–5)	1.1
	4. Communicate the key principles of TCPS2	2.8 (1–4)	3.7 (2–5)	0.9

^a^*n* = 12 attendees participated in the evaluation in the pre-period

^b^*n* = 11 attendees participated in the evaluation in the post-period

### Workshop 1: respect for persons

Workshop 1 was anchored to the core ethical principle “Respect for Persons,” which incorporated the dual moral obligations to respect autonomy, and to “protect those with developing, impaired, or diminished autonomy” [8 p. 6]. Respecting autonomy was presented in relation to obtaining voluntary, informed, and ongoing consent (Ch. 3: “The Consent Process”). Discussion was grounded in the context of the DTES whenever possible. For example, balancing fair compensation with the potential for coercion was discussed with respect to economic marginalization experienced by many DTES residents. Participants were also guided in a critical discussion of the “informed consent form” and considered ways consent can extend beyond a signature on a form (e.g., ongoing consent) [25].

Along with informed consent, workshop 1 also included a discussion of foreseeable research risks and benefits. Drawing from examples of research with people who use(d) illicit drugs, participants were guided in a discussion of social, psychological, behavioral, physical, and economic research risks. For example, the risk for research to re-traumatize participants or cause psychological distress was discussed. Extending conceptualizations of risks and harms beyond the individual, the discussion also considered research harms that can be experienced by communities, with a focus on the potential for exploitation, stigmatization, and community conflict [26].

The concept of developing, impaired, or diminished capacity was discussed in relation to vulnerability, or the ability to safeguard one’s own interests [8]. Attendees were asked to brainstorm situations and research contexts in which people who use(d) illicit drugs may experience vulnerability during research to a greater or lesser degree. The potential for limited access to social goods, rights, opportunities, and power was discussed in relation to vulnerability, while stressing that no individual or group should be considered inherently vulnerable in all circumstances [27].

### Workshop 2: concern for welfare

Workshop 2 focused on “Concern for Welfare” and introduced the *Research 101 Manifesto* [24]. The TCPS2 conceptualizes concern for welfare as the impact of research on an individual’s “physical, mental, and spiritual health as well as physical, economic and social circumstances” [8 p. 7]. We used the case example of a well-known heroin-assisted therapy study in the DTES [28, 29] to introduce the concept of clinical trials (Ch. 11: “Clinical Trials”). The welfare of participants was discussed in relation to not extending heroin-assisted therapy to participants after the trial concluded [28].

Concern for welfare also includes the control of information about participants in research, and workshop 2 introduced concepts of confidentiality and privacy (Ch. 5: “Privacy and Confidentiality”). Privacy is defined as “an individual’s right to be free from intrusion or interference by others” [8 p. 78] and the ethical duty of confidentiality is defined as “the obligation to safeguard entrusted information” [8, p. 78]. Attendees discussed sensitive information that is commonly collected for research with people who use(d) illicit drugs, such as substance use, methods of income generation, and HIV status, and imagined harms that could arise if privacy or confidentiality was compromised. Strategies for safeguarding information, including physical (e.g., lock and key), administrative (e.g., privacy training), and methodological (e.g., using pseudonyms) approaches were described. Brainstorming types of information that are considered identifiable, and how assembling different information can lead to re-identification, rounded out the activity.

Discussion on material from the *Research 101 Manifesto* was led by some of its co-authors with lived and living experience using illicit substances and harm reduction work. Content included an overview of the disproportionate volume of research studies in the DTES [21], the potential and pitfalls of research practices, and principles of community ethics as outlined in the *Research 101 Manifesto* [24]. The four key principles of the *Research 101 Manifesto* included: (1) researcher transparency, or an acknowledgment that researchers in the DTES should be reflexive about their positionality, motivations, and assumptions before developing research partnerships; (2) community-based ethical review, or a call to consider the potential differences between “institutional” ethics (e.g., TCPS2) and “community” ethics [30]; (3) the respectful involvement of peer research assistants and community members through the research process; and (4) reciprocity, or the expectation for researchers to not only return research findings to community, but to also consider how communities can benefit from research [24].

Workshop 2 ended with a case example of a fictitious arts-based body mapping research project about the impact of stigma on people who use(d) illicit drugs in the DTES. The group discussed risks of psychological harms associated with sensitive topics, such as stigma, the secondary use of data, research outputs (e.g., “What should happen to the participants’ body maps after the study is complete?”), and potential threats to confidentiality and re-identification from assembling and presenting a variety of information about an individual.

### Workshop 3: justice

The third and final workshop was anchored to the ethical principle of “Justice,” while also re-visiting the key TCPS2 principles, and summarizing all three workshops’ central takeaways. Justice is conceptualized as “the obligation to treat people fairly and equitably” [8 p. 9], including the just distribution of benefits and burdens of participation in research (TCPS2, Ch. 4: “Fairness and Equity in Research Participation”). The just distribution of benefits and burdens of research was a common theme throughout the three workshops, particularly in relation to the DTES as an “over-researched” community, and the ethical principle of reciprocity (i.e., how communities and researchers can mutually benefit from research) [24]. The workshop also examined inclusion and exclusion criteria in research, including groups (e.g., women, children, older people) who are often excluded from participating in research, and how inclusion and exclusion criteria are implemented for research about substance use (e.g., clinical diagnoses, self-reported behaviors) [28].

Ethical issues related to dual roles and relationships, or perceived conflicts of interest (Ch. 7: “Conflicts of Interest”), were also addressed. Conflicts of interest may impede the autonomous choice of an individual to participate in research [8]. The workshop contextualized the concept of conflicts of interest to CBPR practices and the involvement of peer research assistants. Attendees described the benefits, and ethical challenges, of being an “insider” within a community. The workshop concluded with a case example of a fictitious qualitative research study about frontline harm reduction work experiences, in which peer research assistants with experience as harm reduction workers participated in study recruitment, data collection, and analysis. Attendees brainstormed the dual roles they may hold (e.g., as harm reduction workers and as peer research assistants) and discussed opportunities to avoid and manage conflicts of interest.

## Discussion

Community-based participatory research can involve non-academic community members in roles that impact study safety and integrity [18]. People with lived and living experience are commonly employed as peer research assistants and may be involved in study recruitment, data collection, and analysis [1]. In our experience, these practices raise an institutional requirement for training in research ethics, although existing approaches are largely inaccessible or inappropriate for non-academic team members. In this article, we described an alternative approach to research ethics training that we deployed for a CBPR project in Vancouver’s DTES with people who use(d) illicit drugs and harm reduction workers. We collaborated with our institutional REB to ensure that our workshops met necessary institutional requirements. Evaluation results suggest that the workshops were acceptable, and contributed to increased confidence in learning objectives among attendees. Our approach may serve as a model that could be adopted for CBPR with people who use(d) drugs and harm reduction workers in other contexts.

Although ethical principles for the responsible conduct of research outlined in the TCPS2 and Belmont report aim to guide researchers in ethical decision-making, “ethically important moments” often occur in the field [16, 31]. Situating research ethics training within a particular research context can help prepare study team members to identify and respond to ethical issues as they arise in practice [32]. The CERET workshops integrated tailored case studies and illustrative examples, equipping attendees to identify and attend to ethical issues that might arise throughout research with people who use(d) illicit drugs and harm reduction workers.

Along with providing much-needed context for applying ethical principles in research with people who use(d) illicit drugs, the CERET workshops also attended to dimensions of research ethics not addressed in guidelines for the ethical conduct of research. Community-based researchers have long advocated for broadened conceptualizations of research ethics beyond procedural ethics, and challenged how academic institutions have defined what “counts” as relevant dimensions of research ethics [14, 30]. Schienke et al. [32] have characterized three overlapping dimensions of research ethics: “procedural,” “intrinsic,” and “extrinsic” ethics. Traditional research ethics training and institutional processes have focused primarily on “procedural” ethics (i.e., guidelines for the ethical conduct of research and review procedures), while neglecting “intrinsic” and “extrinsic” ethical issues.

“Intrinsic” ethical issues are internal to a mode of inquiry (e.g., a given research approach) and arise from values and assumptions embedded in scientific practice [32]. The CERET workshops attended to intrinsic ethical issues by integrating content from the *Research 101 Manifesto,* which calls on researchers to reflect on their positionality, privilege, and assumptions, and consider how these factors relate to motivations for conducting the research, or selecting a research question. These factors are representative of ethical values and choices embedded within research. The *Research 101 Manifesto* also calls on researchers working in the DTES to consider how community members can be integrated respectfully throughout the research process. For example, ethical issues that arise from involving peer research assistants were discussed throughout the CERET workshops.

“Extrinsic” ethical issues are those external to knowledge production and consider the societal impacts of scientific research [32]. Practitioners of CBPR are well-equipped to address extrinsic ethical issues, as CBPR is fundamentally defined by its relationship to community [33]. Discussion of extrinsic ethical issues in CERET workshops centered around concepts like research risks and reciprocity. Guidelines for the ethical conduct of research, including the TCPS2 [13], predominantly operate within a biomedical framework focused on risks to individuals, and overlooking risks posed to communities [14]. Following Ross et al. [26] the CERET workshops viewed community level risks as including the potential for research findings to stigmatize social identities and communities, and to disrupt community cohesion or undermine the group’s moral or sociopolitical authority.

On the other hand, the potential for communities to benefit from research was presented as the ethical principle of reciprocity. The *Research 101 Manifesto* operationalized reciprocity as not only the ethical imperative for research findings to be returned to community, but also as a call for researchers to consider how communities can benefit from research [24]. Reciprocity is an extrinsic ethical issue, because it requires that researchers take part in reflexive and anticipatory thinking about how their research activities contribute to the goals and values of the communities in which they operate [32]. Despite being often over looked in traditional research ethics training, intrinsic and extrinsic ethical issues are of ethical and epistemological significance, and are relevant to all community partners in research, regardless of their direct involvement in roles that impact study safety and integrity.

One limitation of our approach to research ethics training was that it was relatively resource-intensive. As Jetter et al. [20] describe, true partnerships between academic partners and communities require significant time and resources. We added new content beyond what was institutionally required in order to attend to the broad spectrum of procedural, intrinsic, and extrinsic ethical issues that arise in the context of CBPR. Furthermore, to make the CERET workshops accessible, we delivered workshops in person, over multiple days, and provided food and honoraria. The discussion-based format allowed for a bi-directional exchange of knowledge and recognized that all attendees had unique, relevant expertise across the ethical dimensions of research. Four workshop participants were co-authors of the *Research 101 Manifesto*, and co-facilitated portions of the workshops. Ultimately, we felt that taking time to meaningfully build capacity for research ethics was most in line with our CBPR practice, and would help empower non-academic community members to make ethical decisions throughout the project.

Our approach to community-engaged research ethics training differs from what we have observed in some practices, where academic researchers request that non-academic community members be exempt from research ethics training, not listed on ethics applications, or relegated to a nebulous list of “research personnel” for whom research ethics training may not be required. Although these practices may be understandable in the absence of accessible alternatives, we believe approaches that promote capacity-building in ethics can encourage greater involvement of community in all dimensions of research processes, including arenas of knowledge production often considered to be the purview of academic experts and institutional gatekeepers. Ultimately, this involvement can strengthen the integrity of the research process, by integrating ethical perspectives beyond those of academic team members.

## Conclusion

Community-based participatory research often involves community members in roles that can necessitate training in research ethics. Existing approaches to research ethics training, often accomplished through self-guided online tutorials, can be inaccessible to study team members without an academic background, and can be experienced or perceived as a barrier to community engagement in research [11, 16]. Additionally, standardized trainings address a relatively narrow set of ethical issues, mostly related to guidelines for the ethical conduct of research (e.g., TCPS2, Belmont report), and may neglect ethical issues most relevant to CBPR [13, 14]. The CERET workshops offered an approach to fulfill an institutional requirement for training in research ethics, and meaningfully build capacity with people who use(d) illicit drugs and harm reduction workers.

In alignment with principles of CBPR, building capacity in research ethics recognizes and substantiates community members as ethical agents and partners in ethical decision-making throughout the research process. While in some cases, community members may be directly involved in study roles with clear implications for participant safety and data integrity, ethical dimensions of research and ethically important moments occur throughout the research process. Even when community members are not engaged in capacities that necessitate any institutional requirement for research ethics training, building capacity around the intrinsic and extrinsic dimensions of research can prepare all study team members to attend to ethical issues that arise from CBPR.

## Data Availability

Workshop presentation slides, facilitator notes, and a facilitator’s guide are available from the corresponding author upon request. Data generated from the evaluation of workshops are not publicly available due to privacy concerns related to the small sample size.

## Abbreviations

CBPR: Community-based participatory research
TCPS2: Tri-Council Policy Statement: Ethical Conduct for Research Involving Humans
CORE: Course on research ethics
CITI: Collaborative Institute Training Initiative
US: United States
REB: Research ethics board
CERET: Community-Engaged Research Ethics Training
DTES: Downtown Eastside
OPPRA: Overdose Prevention Peer Research Assistant
